# Attention bias modification for depression: A systematic review and meta-analysis

**DOI:** 10.3389/fpsyt.2023.1098610

**Published:** 2023-03-10

**Authors:** Hai-sha Xia, Yu-xi Li, Qing-yun Zhang, Dong-ling Zhong, Xiao-bo Liu, Xin-yun Gou, Jin Fan, Jing Zhao, Yue Zhang, Shuang-chun Ai, Jia-xi Huang, Juan Li, Rong-jiang Jin

**Affiliations:** ^1^School of Health Preservation and Rehabilitation, Chengdu University of Traditional Chinese Medicine, Chengdu, China; ^2^Department of Rehabilitation, The Third Hospital of Mianyang, Sichuan Mental Health Center, Mianyang, China; ^3^Department of rehabilitation, Mianyang Hospital of Traditional Chinese Medicine, Mianyang, China; ^4^Mental Health Center, West China Hospital, West China School of Medicine, Sichuan University, Chengdu, China

**Keywords:** depression, cognitive deficits, attention bias modification, systematic review, meta-analysis

## Abstract

**Background:**

Depression is a mental health disorder characterized by affective, somatic, and cognitive symptoms. Attention bias modification (ABM) has been widely used to treat depression. However, the results seem inconsistent. We conducted a systematic review and meta-analysis to investigate the efficacy of ABM for depression and to explore the optimal protocol of ABM.

**Methods:**

Seven databases were systematically searched from their inceptions to 5 October 2022 to include randomized controlled trials (RCTs) of ABM for depression. Two independent reviewers selected the eligible articles, extracted data, and evaluated the risk of bias using version 2 of the Cochrane risk-of-bias tool (ROB 2.0) for randomized trials. The primary outcome was the evaluation of depressive symptoms using widely accepted and validated scales. The secondary outcomes included rumination and attentional control. Meta-analysis was conducted by using RevMan (version 5.4) and Stata (version 12.0). Subgroup analyses and meta-regressions were performed to identify the source of heterogeneity. The certainty of the evidence was assessed using the Grading of Recommendations Assessment, Development, and Evaluation (GRADE).

**Results:**

A total of 19 trials involving 20 datasets (1,262 participants) were included. The overall risk of bias in one study was rated as low risk of bias, three studies were considered as high, and the remaining studies were some concerns. Compared with attention control training (ACT), ABM had a greater effect in the improvement of depression (SMD = −0.48, 95% CI −0.80 to −0.17, *I^2^* = 82%) and rumination (MD = −3.46, 95% CI −6.06 to −0.87, *I^2^* = 0%). No significant differences were observed in the attentional control outcome between ABM and ACT (MD = 3.07, 95% CI −0.52 to 6.65, *I*^2^ = 0%). Subgroup analysis demonstrated that adults exhibited a greater decrease in depression scores than adolescents. ABM using the dot-probe task, training target stimulus presented by face, and training directions by left–right were associated with better antidepressant effects. ABM training delivered in the laboratory tended to yield a better effect than those conducted at home. Sensitivity analysis indicated that the results were robust. The certainty of the evidence for all outcomes was low or very low, and publication bias may exist.

**Conclusion:**

Due to high heterogeneity and limited studies, not enough current evidence supported that ABM could be an effective intervention to relieve depressive symptoms. More rigorous RCTs are required to verify the benefits and to explore the optimal protocol of ABM training for depression.

**Systematic Review Registration:** [PROSPERO], identifier [No. CRD42021279163].

## 1. Introduction

Depression is a common mental disorder characterized by a persistent low mood and anhedonia, with an approximately 16% lifetime prevalence (1) and is affecting nearly 350 million individuals (2). Since the outbreak of the COVID-19 pandemic in 2019, 52 million new major depressive disorder cases had been diagnosed globally, with an increase of 27.6% (3). In the United States, the absence days from work due to depression were estimated to be 27.2 workdays per patient with depression a year (4), which brought a significant financial burden to patients, families, and society (5). According to the cognitive theory of depression, the acquisition and processing of information are considered to be significant contributors to the occurrence and development of depression (6). Individuals with depression are unable to process all sensory information equally, and they selectively tend to focus on negative emotional information (7, 8). Negative attentional bias and deficits in cognitive control may interfere with emotion regulation and mood state. The increased activation of subcortical emotion processing regions and a weakening of top-down cognitive control may be responsible for negative cognitive biases (9). At present, attention bias modification (ABM) for depressive individuals has attracted increasing attention.

As a type of cognitive bias modification, ABM utilizes computer-based attention training to directly modify aberrant attentional bias in patients with depression (10). ABM aims at increasing the process of neutral or positive stimulation to reduce negative attentional bias, thus regulating emotional function (11, 12). ABM relies on the automatic cognitive processing of altering motivation, rather than solely changing the content of individual behaviors (13–15). In recent years, several paradigms of ABM have been devised and applied, which include the dot-probe task (DPT), the spatial cueing task (SCT), and the free viewing task (FVT) (16). Clinical studies showed that ABM was able to reduce depressive symptoms in situations when negative attentional bias was successfully modified (17, 18). Therefore, ABM programs could be a promising treatment for depressive symptoms. In addition, considerable evidence indicated that ABM had a positive effect on other psychiatric disorders such as anxiety disorders (10, 19), social phobia (20), and obsessive–compulsive disorders (21, 22).

Previous meta-analyses (23–25) concluded that ABM was not effective for patients with depression. However, Yang et al. (17) found that ABM had a significant effect to decrease BDI scores when compared with the placebo condition. Woolridge et al. (26) discovered that ABM might be an optimal treatment to relieve depressive symptoms. Furthermore, the optimal protocols of ABM (e.g., task types, target stimulus, stimulus directions, and training settings) for depression remain unknown. As more relevant trials have been conducted in recent years, we performed this systematic review (SR) and meta-analysis to update the evidence on the effect of ABM on depression and to explore the optimal protocols of ABM.

## 2. Methods

The protocol of this SR and meta-analysis has been registered on the International Prospective Register of Systematic Reviews (PROSPERO).^1^ We conducted this SR and meta-analysis according to A Measurement Tool to Assess Systematic Reviews 2 (AMSTAR 2) (27) and reported conforming to the preferred reporting items for systematic reviews and meta-analyses (PRISMA 2020) statement criteria (28) (Supplementary Appendix 1).

### 2.1. Search strategy

Two reviewers independently (HSX and XYG) searched PubMed, Embase, the Cochrane Library, Chinese National Knowledge Infrastructure (CNKI), Wanfang Database, Chinese Biomedical Literature Database (CBM), and China Science and Technology Journal Database (VIP) from their inceptions to 5 October 2022. Search terms used depression, attention bias modification, and randomized controlled trial. The full search strategies for all databases are shown in Supplementary Appendix 2. We manually searched the reference lists of all identified articles, gray literature, and relevant registration websites^2^ for possible eligible studies. In addition, we consulted the relevant experts for potential studies.

### 2.2. Inclusion criteria

Studies were included if they fulfilled all the inclusion criteria: (1) Patients diagnosed with depression based on the Diagnostic and Statistical Manual of Mental Disorders (DSM) (29), International Classification of Diseases (ICD) (30), Chinese Classification and Diagnosis of Mental Diseases (CCMD), or validated scales (24, 31). There were no restrictions on race, gender, or age. (2) Intervention included ABM alone, or ABM plus conventional treatment (CT). CT contained medication and psychological intervention. There were no limitations on task types, stimulus types, and training directions of ABM. (3) Participants in the control group received attention control training (ACT) alone, ACT plus CT, or CT alone. (4) The primary outcome was depressive symptoms evaluated with widely accepted and validated scales. Secondary outcomes included rumination and attentional control. (5) RCTs that investigated the effect of ABM on patients with depression were included.

### 2.3. Exclusion criteria

Studies were excluded if they met any of the following criteria: (1) studies using interpretation bias modification; (2) cross-over RCTs, review articles, and conference abstracts; (3) overlapping publications; (4) the full texts were not available through various approaches.

### 2.4. Study selection

Endnote X9 was used to manage the retrieved records. After removing duplicates, two independent reviewers (H-sX and X-bL) screened the titles and abstracts to identify the potential studies. Then, the rest records were scrutinized in full text. Any inconsistency was resolved through consultation with the third reviewer (JL). If multiple publications reported data from the same trial, we included the article with the most complete or latest data.

### 2.5. Data collection and extraction

Two independent reviewers (H-sX and X-yG) extracted data from included studies with a standard extraction form. The following data were extracted: (1) study information: first author, publication year, and country; (2) participant characteristics: diagnostic criteria, sample size, and age; (3) details of interventions: types, paradigms, stimulus types, stimulus directions, sessions, and total trials of per session; (4) comparators: types of intervention, frequency, and duration; (5) primary outcome and secondary outcomes; (6) information related to the risk of bias. With regards to missing data, corresponding authors were contacted *via* email for missing or incomplete data. For multi-arm RCTs, we extracted the eligible comparisons or the comparison with an inferior effect size. If the data was displayed in the graph, the GetData Graph Digitizer 2.26 was used to extract the data. After cross-checking, disagreements were settled through consultation with an experienced reviewer (Y-xL).

### 2.6. Risk-of-bias assessment

Two researchers (JF and D-lZ) separately evaluated the risk of bias using version 2 of the Cochrane risk-of-bias tool for randomized trials (RoB 2.0). There are five domains in RoB 2.0: randomization process, deviations from intended interventions, missing outcome data, measurement of the outcome, and selection of the reported results. Each domain is rated as “low risk of bias,” “some concerns,” or “high risk of bias.” In case of disagreements, a third investigator (JL) was involved.

### 2.7. Certainty of The evidence

The Grading of Recommendations Assessment, Development, and Evaluation (GRADE) system was used to assess the certainty of the evidence of each outcome (32). Each outcome was evaluated from the following five aspects: limitations, inconsistency, indirectness, imprecision, and publication bias. The certainty of the evidence was categorized as “high,” “moderate,” “low,” or “very low.” (33).

### 2.8. Statistical analysis

Data synthesis was conducted using RevMan (version 5.4) and Stata (version 12.0). Among the included studies, different measurement tools were used to evaluate the symptoms of depression, and the standardized mean difference (SMD) was calculated (34–36). The ability of attention control among the included studies was evaluated using an attention control scale (ACS), and the ruminative symptoms were assessed with a ruminative response scale (RRS); thus, weighted mean difference (WMD) was used to synthesize these results. The uncertainty was expressed with 95% confidence intervals (CIs). The statistical heterogeneity across the included studies was assessed by the Chi-squared test and *I*^2^ statistic. The fixed-effect model was performed when *p* > 0.1 or *I*^2^ values < 50%. Otherwise, the random-effect model was used. Forest plots were used to display the pooled estimates, and a value of *p* < 0.05 was regarded as statistically significant. If the pooled data could not be synthesized, we conducted the descriptive analysis.

### 2.9. Subgroup analysis and meta-regressions

Subgroup analyses were conducted according to age, task types, training target stimuli, training directions, and training settings, whereas meta-regressions were performed based on BDI scores at baseline, publication year, gender, number of training sessions, and number of training trials per session.

### 2.10. Sensitivity analysis

The sensitivity analysis was conducted by eliminating studies with a high risk of bias to verify the robustness of the results.

### 2.11. Publication bias

The funnel plot was used to assess possible publication bias when ≥10 studies were included in the analysis.

## 3. Result

### 3.1. Selection and inclusion of studies

**Figure 1:**
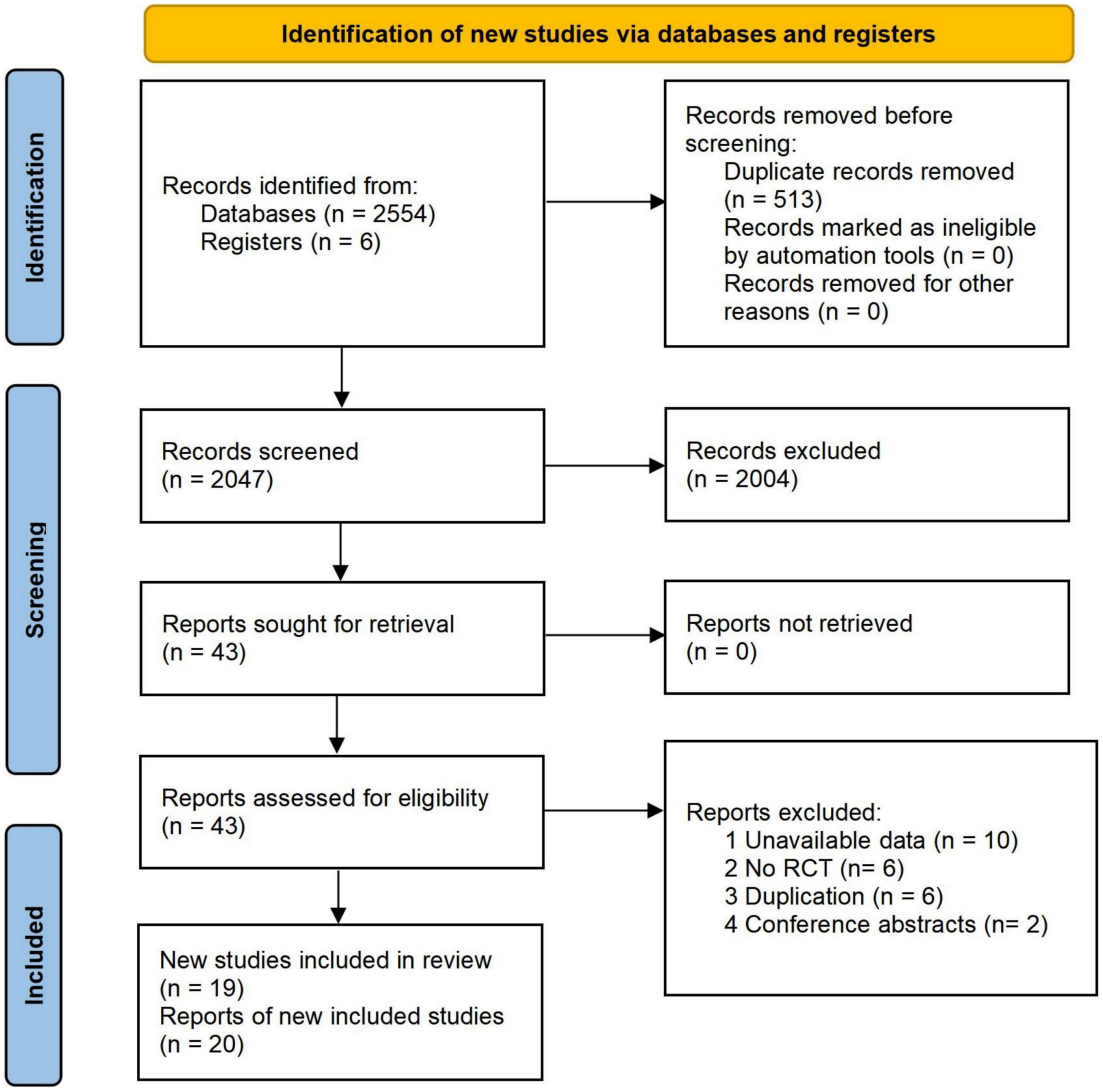
Preferred reporting items for systematic reviews and meta-analyses (PRISMA) flow chart of literature searching and screening.

A total of 2,560 articles were identified. After removing 513 duplicates, 2,004 articles did not conform with the eligible criteria and were excluded. Among the remaining 43 records, 24 studies were excluded after reviewing the full text. Then, we included 20 datasets from 19 reports. The diagram of the screening process is shown in Figure 1. The list of excluded records with reasons is provided in Supplementary Appendix 3.

### 3.2. Characteristics of included studies

The characteristics of the included trials are shown in Table 1. A total of 19 trials involving 20 datasets with 1,262 patients with depression were included, wherein Baert et al. (37) observed the effect of ABM for patients with depression diagnosed by DSM-IV criteria and BDI-II scales separately; therefore, we extracted these two datasets, respectively. Among included studies, nine studies were performed in China (17, 18, 39, 43, 44, 48, 49, 51, 52), two were in Belgium (37), two were in the United States (41, 50), two were in the United Kingdom (45, 46), and one in Norway (38), Netherlands (40), Poland (42), Israel (47), and Canada (26). The sample size of these studies varied from 30 to 301. The age of the included patients ranged from 14 to 45. Sixteen studies involved adults (17, 26, 37–39, 41, 42, 44–50, 52), and four studies included adolescents (18, 40, 43, 51). Among included studies, four studies were three-arm trials (17, 40, 41, 48), and the rest studies were two-arm trials. As for comparison, 17 studies compared ABM with ACT (17, 18, 26, 37–42, 45–48, 50–52), two studies compared ABM plus CT versus ACT plus CT (43, 49), and only one study compared ABM plus CT versus CT (44). The number of training sessions ranged from 1 to 28, and the duration of training was between 1 and 4 weeks.

**Table 1:** Characteristics of included studies.

Study	Country	Diagnostic criteria	Sample size (R/A)	Sample size (E/C)	Mean age (years)	Gender ratio	Intervention							Comparison	Duration	Outcomes
						(F/M)	Type	Paradigm	Stimulus types	Training directions	Sessions	Total trials of per session	Training settings	Type		
Baert 2010a (37)	Belgium	BDI-II ≥ 19	48/48	E: 25	E: 19.88	F: 44	ABM	SCT	Positive/neutral/negative words	Left–Right	10	220	Home	ACT	1×/day for 10 days	BDI-II RRS
				C: 23	C: 20.09	M: 4										
Baert 2010b (37)	Belgium	DSM-IV/	35/35	E: 15	E: 39.87	F: 22	ABM	SCT	Positive/neutral/negative words	Left–Right	10	220	Home	ACT	1×/day for 10 days	BDI-II RRS
		MINI		C: 20	C: 46.3	M: 13										
Bø	Norway	MINI	301/301	E: 153	E: 40.2	F: 212	ABM	DPT	Positive/neutral/	Top-Bottom	28	96	Lab	ACT	2×/day for 2 weeks	HDRS
2021 (49)				C: 148	C: 41.5	M: 89			negative faces							
Dai 2019 (38)	China	DSM-IV	32/32	E: 16	E: 38.31	F: 18	ABM	SCT	Positive/neutral/negative faces	Left–Right	10	480	Lab	ACT	1×/day for 10 days	HDRS
				C: 16	C: 39	M: 14										
De Voogd 2017 (50)	Netherlands	SCARED>16/CDI > 7	108/70	E: 32	E: 14.73	F: 72	ABM	VST	Positive/negative faces	NR	8	36	Home	C1: ACT	2×/week for 4 weeks	CDI
				C1: 26	C1: 14.31	M: 36								C2: NT		
				C2: 36	C2: 14.29											
Hsu 2021 (45)	The United States	QIDS-SR ≥ 13	145/116	E: 38	E: 24.4	F: 111	ABM	DPT	Positive/neutral/negative faces	Left–Right	20	Lab: 198/	Lab and home	C1: ACT	5×/week for 4 weeks	HRSD
				C1: 38	C1: 25.3	M: 34						Home: 66		C2:NT		
				C2: 40	C2: 26.1											
Krejtz 2018 (51)	Poland	DSM-IV	60/51	E: 26	E: 36.12	F: 34	ABM	DPT	Positive/neutral faces/words/images	Top-Bottom	14	90	Lab	ACT	1×/day for 2 weeks	CES-D
				C: 25	C: 33.96	M: 17										
Liao 2016 (39)	China	DSM-IV	86/86	E: 43	E: 14.39	F: 51	ABM + CT	DPT	Neutral/negative words	NR	NR	160	Lab	ACT+CT	4 weeks	HAMD
				C: 43	C: 14.36	M: 45										
Liu 2018 (40)	China	ICD-10	60/53	E: 26	E: 37.38	F: 26	ABM + CT	SCT	Neutral/negative words	Top-Bottom	12	320	Lab	CT	3×/week for 4 weeks	HAMD
				C: 27	C: 36.81	M: 27										
Penton-Voak 2012 (47)	The United Kingdom	BDI-II ≥ 14	80/75	E: 37	E: 21	F: 55	ABM	FVT	Positive/neutral/negative faces	Randomly	4	186	Lab	ACT	1×/day for 4 days	BDI-II
				C: 38	C: 21	M: 25										
Penton-Voak 2021 (48)	The United Kingdom	DSM-IV/	36/36	E: 19	E: 21	F: 24	ABM	FVT	Positive/neutral/negative faces	Randomly	5	186	Lab	ACT	1×/day for 4 days	BDI-II
		BDI-II ≥ 14		C: 17	C: 23	M: 12										
Shamai- Leshem 2021 (52)	Israel	MINI	60/47	E: 25	E: 43.37	F: 26	ABM	FVT	Positive/negative faces	Randomly	8	60	Lab	ACT	2×/week for 4 weeks	BDI-II
				C: 22	C: 40.33	M: 34										
Wang 2018 (41)	China	BDI-II ≥ 13	73/65	E1: 20	E1: 19.2	F: 51	E1: Positive ABM	DPT	Positive/neutral/negative faces	Left–Right	8	168	Lab	ACT	2×/week for 4 weeks	BDI-II ACS
				E2: 21	E2: 18.86	M: 14	E2: Neutral ABM									
				C: 24	C: 19.54											
Wei 2020 (42)	China	CES-D>20	68/68	E: 34	E: 34.2	F: 33	ABM + CT	DPT	Neutral/negative words	NR	NR	NR	Lab	ACT+CT	24 weeks	HAMD
				C: 34	C: 35.8	M: 35										
Wells 2010 (46)	The United States	BDI-II ≥ 9	34/31	E: 14	19.1	NR	ABM	DPT	Neutral/negative faces	Left–Right	4	196	Lab	ACT	2 weeks	BDI-II
				C: 17												
Woolridge 2021 (26)	Canada	MINI	46/40	E: 20	E: 44.9	F: 26	ABM	FVT	Neutral/negative words	Randomly	3	168	Lab	ACT	1 week	BDI-II
				C:20	C: 44.15	M: 14										
Yang 2015 (17)	China	DSM-IV	77/77	E: 27	E: 19.44	F: 55	ABM	DPT	Neutral/negative words	Top-Bottom	8	108	Lab	C1: ACT	4×/week for 2 weeks	BDI-II RRS
				C1: 27	C1: 19.52	M: 22								C2:NT		
				C2: 23	C2: 19.57											
Yang 2016 (18)	China	DSM-IV	45/45	E: 23	E: 15.09	F: 25	ABM	DPT	Positive/neutral/negative words	Top-Bottom	8	320	Lab	ACT	4×/week for 2 weeks	HAMD RRS
				C: 22	C: 14.82	M: 20										
Zheng 2018 (43)	China	DSM-V	30/30	E: 15	E: 17.8	F: 25	ABM	DPT	Neutral/negative words	Top-Bottom	13	108	Lab	ACT	3 weeks	BDI-II RRS ACS
				C: 15	C: 18.2	M: 5										
Zhou	China	CCMD-3	40/40	E: 20	E: 20.13	NR	ABM	DPT	Neutral/negative faces	Top-Bottom	12	200	Lab	ACT	3×/week for 4 weeks	BDI-II
2017 (44)				C: 20	C: 20.65											

DSM-IV/V: Diagnostic and Statistical Manual of Mental Disorders, Fourth/Fifth Edition; ICD-10: The International Classification of Diseases, Tenth Edition; MINI: Mini International Neuropsychiatric Interview; CCMD-3: Chinese Classification and Diagnosis of Mental Diseases, Third Edition; R/A: Randomized/analyzed; E/C: experimental group/control group; F/M: female/male; SCT: spatial cueing task; DPT: dot-probe task; VST: visual search task; FVT: free viewing task; NR: no report; CT: conventional treatment (medication and psychological intervention, etc.); ACT: attention control training; NT: no training d: day; w: week; m: month; BDI-II: Beck Depression Inventory II; HAMD/HDRS/HRSD: Hamilton Depression Rating Scale; CES-D: Center for Epidemiological Studies-Depression Scale; CDI: Children’s Depression Inventory; STAI-T: State and Trait Anxiety Inventory-Trait; BAI: Beck Anxiety Inventory; SCARED: Screen for Child Anxiety Related Emotional Disorders; RRS: Ruminative Responses Scale; ACS: Attention Control Scale.

### 3.3. Risk of bias In studies

**Figure 2:**
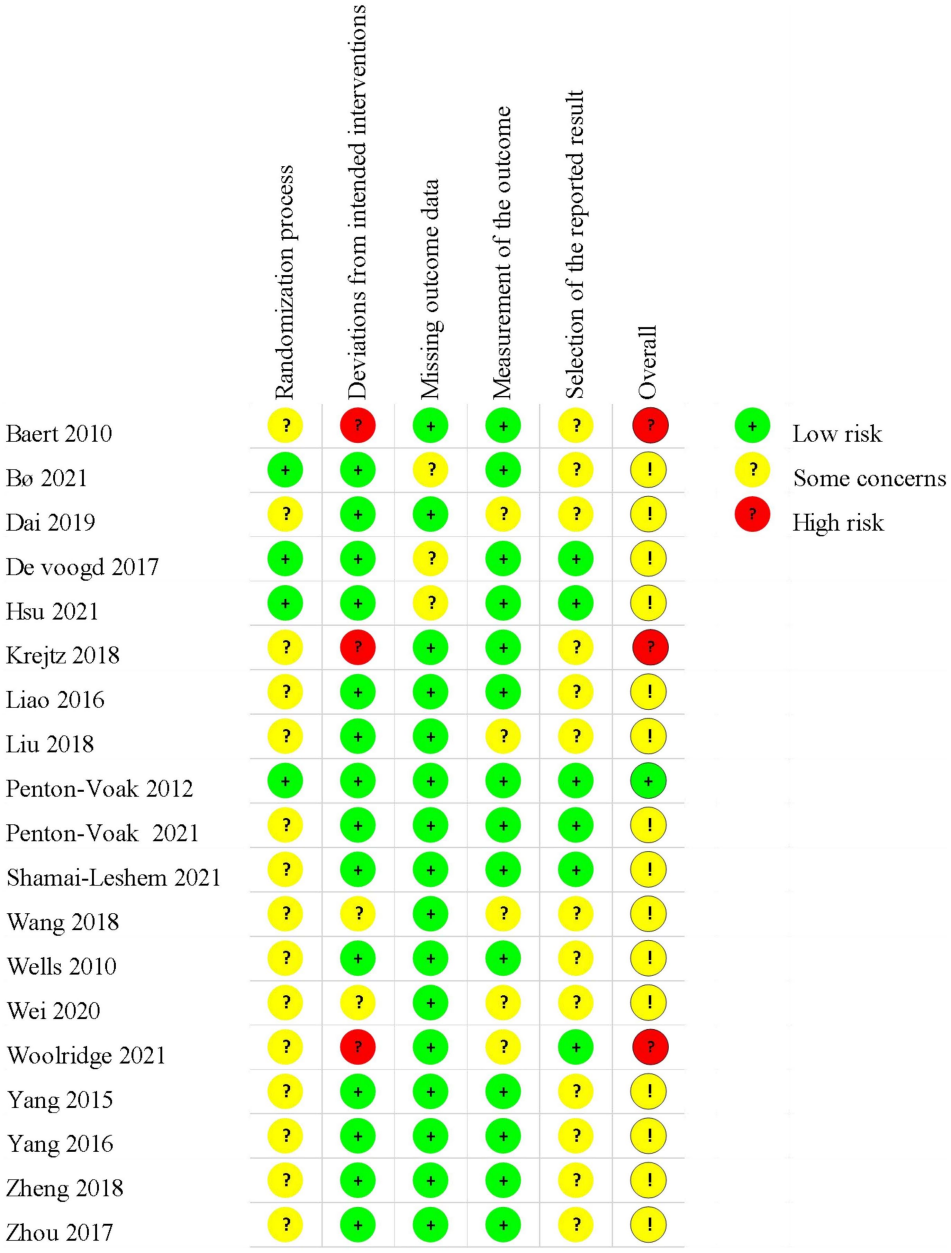
Results of risk-of-bias (RoB 2.0) assessment. The plot of RoB 2.0 for each included study.

**Figure 3:**
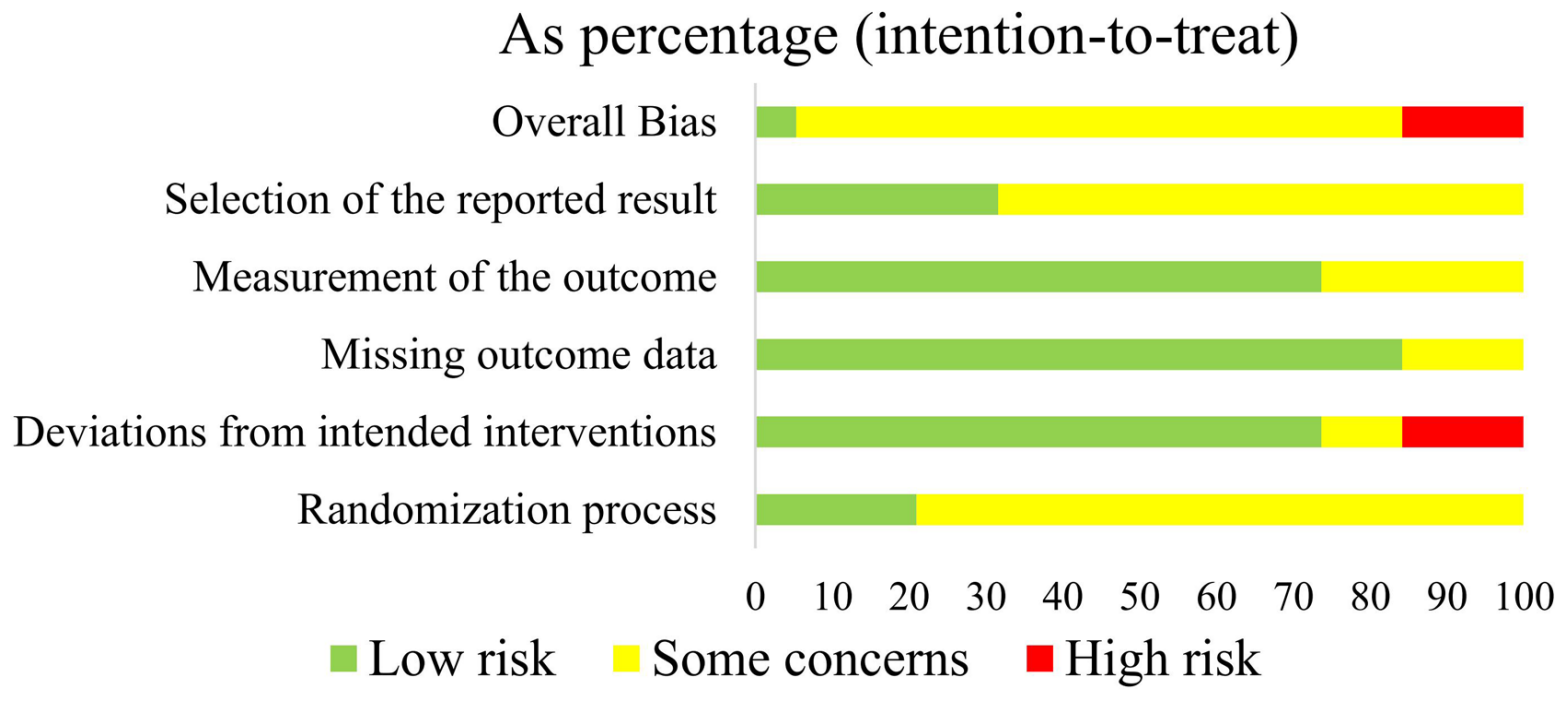
Results of risk-of-bias (RoB 2.0) assessment. Proportions of individual study for each domain.

The plot of the risk of bias (RoB 2.0) for each included study is presented in Figure 2, and the proportions of individual studies are presented in Figure 3.

In the randomization process, all included studies showed no statistically significant difference between groups at baseline. Four studies (38, 40, 41, 45) were judged as low risk, while the rest 15 studies were assessed as having some concerns due to no details of randomization or allocation concealment.

Considering the deviation from intended interventions, 14 trials (17, 18, 38–43, 45–47, 50–52) performed appropriate analysis on all randomly assigned participants, which were judged as low risk of bias. Two studies (48, 49) did not report blinding, which was considered as some concerns. The remaining three trials (26, 37, 44) were regarded as high risk due to no blinding in outcome assessors and inappropriate analysis.

As for the missing outcome, 13 studies (17, 18, 26, 37–42, 44, 45, 47, 51) reported the number of drop-outs or lost to follow-up. Among these studies, three studies (38, 40, 41) did not report the details of drop-outs, which were rated as some concerns.

With regard to the measurement of outcomes, six studies (26, 37, 39, 44, 48, 49) were assessed as some concerns due to the lack of a blinding method of outcome assessors. The remaining 13 studies were a low risk of bias.

For the selection of the reported results, six trials (26, 40, 41, 45–47) provided protocol information and reported most of the expected outcomes comprehensively, which were considered as low risk. The rest trials did not provide protocol information, which was assessed as some concerns.

In summary, the overall risk of bias in one trial was considered as low risk, three trials were considered as high risk, and the remaining were considered as some concerns.

### 3.4. Results of the meta-analysis

#### 3.4.1. Primary outcome (depression)

##### 3.4.1.1. ABM versus ACT

**Figure 4:**
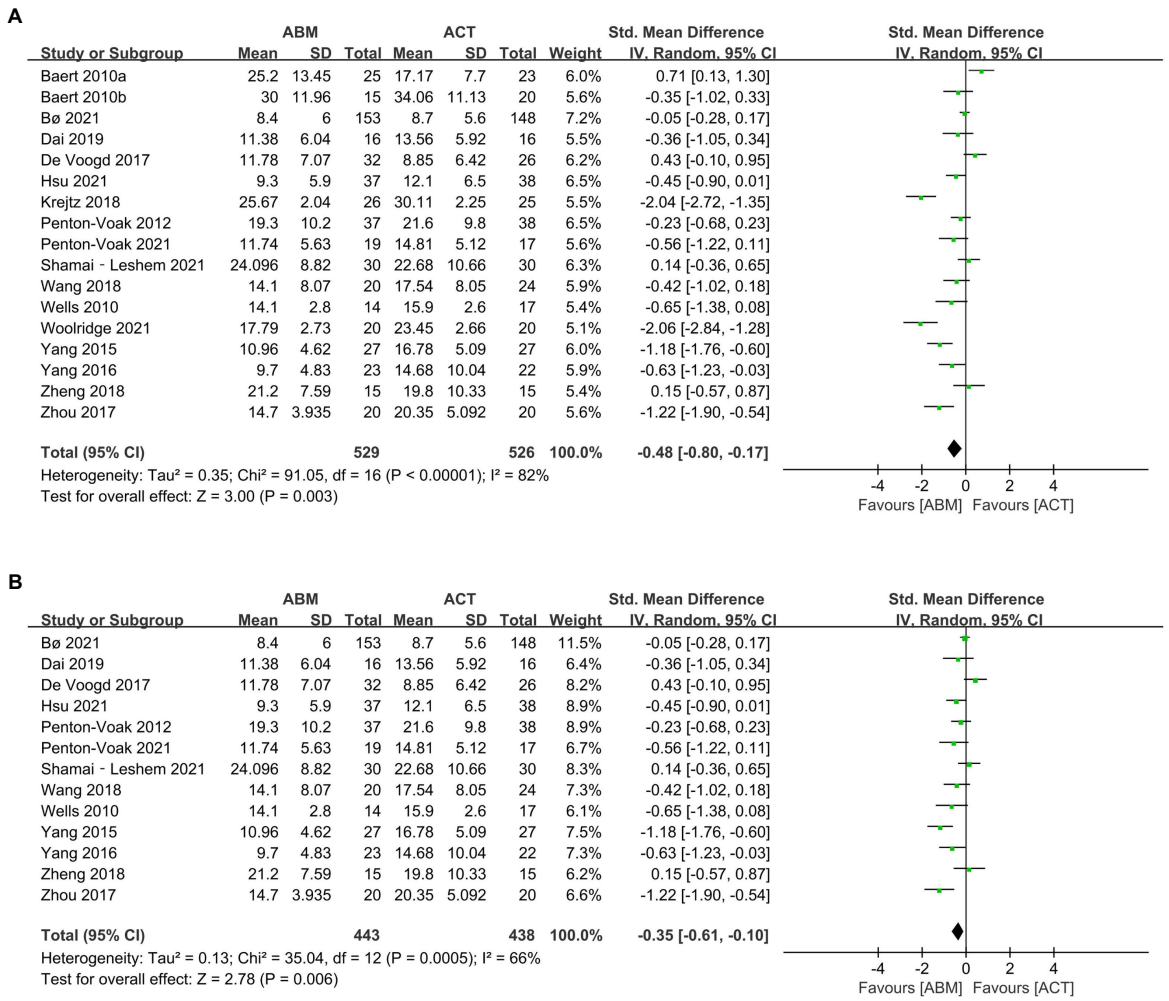
**(A)** Forest plot of depression outcome in comparison with attention bias modification (ABM) versus attention control training (ACT). **(B)** Forest plot of depression outcome in comparison with ABM versus ACT after removing high risk-of-bias studies.

A total of 16 trials (17, 18, 26, 37–42, 45–48, 50–52) involving 17 datasets reported depressive symptoms. The results demonstrated that ABM was superior to ACT in reducing depressive symptoms (SMD = −0.48, 95% CI −0.80 to −0.17, *I*^2^ = 82%; Figure 4A). By exploring heterogeneity, we found the risk of bias in Baert et al. (37), Krejtz et al. (42), and Woolridge et al. (26) were high, while the risk-of-bias assessment in other studies was identified as low risk or some concerns. After removing these datasets (26, 37, 42) with a high risk of bias, sensitivity analysis showed that the overall effects did not change (SMD = −0.35, 95% CI −0.61 to −0.10, *I^2^* = 66%; Figure 4B).

###### 3.4.1.1.1. Subgroup and meta-regression analysis

As depicted in Table 2, the subgroup analysis showed that adults had greater improvement in depression than adolescents. Regarding types of task, ABM using dot-probe task was more effective to relieve depressive symptoms than the ACT, while ABM with spatial cueing, visual search, or free viewing task had no effect. As for the training target stimuli, ABM using face stimulus had a significantly larger effect than those using word stimulus. Concerning training directions, ABM with left–right training alleviated more depressive symptoms than the ACT, while those presented by top-bottom training did not. In addition, the training delivered in the laboratory tended to yield a better effect than those conducted at home.

**Table 2:** Subgroup analyses of depression outcome in comparison with ABM versus ACT.

Subgroup	Number of studies	Patients (E/C)	Overall effect		Heterogeneity	
			Effect size (95% CI)	*p*	*I^2^*	*p*
1.1 Subgroup analysis by different age group						
Adolescents	3	70/63	−0.01[−0.66, 0.64]	0.97	71%	0.03
Adults	10	374/243	−0.46[−0.73,−0.19]	0.0007	55%	0.02
1.2 Subgroup analysis by task types						
Dot-probe Task	8	310/311	−0.53[−0.87, −0.18]	0.003	71%	0.001
Spatial cueing task	1	16/16	−0.36[−1.05, 0.34]	0.32	–	–
Free-viewing task	3	86/85	−0.17[−0.54, 0.19]	0.35	29%	0.25
Visual search task	1	32/26	0.43[−0.10, 0.95]	0.11	–	–
1.3 Subgroup analysis by target stimuli						
Words	3	65/64	−0.58[−1.30, 0.15]	0.12	75%	0.02
Faces	10	379/374	−0.28[−0.53, −0.03]	0.03	59%	0.009
1.4 Subgroup analysis by training directions						
Top-Bottom	5	238/232	−0.57[−1.12, −0.01]	0.05	83%	0.0001
Left–Right	4	87/95	−0.46[−0.75, −0.16]	0.002	0%	0.95
1.5 Subgroup analysis by training settings						
Lab	12	375/374	−0.42[−0.69, −0.15]	0.002	64%	0.002
Home	1	32/26	0.43[−0.10, 0.95]	0.11	–	–

We performed meta-regression in accordance with gender (percentage of females; range 43.3–83.3%), publication year (range 2010–2021), BDI at baseline (range 17.1–29.94 scores), number of training sessions (range 4–28 sessions), and number of training trials per session (range 36–480 trials). The results indicated that BDI at baseline was a moderator of the ABM, lower BDI at baseline benefited more from ABM (Table 3).

**Table 3:** Meta-regression of depression outcome in comparison with ABM versus ACT.

Moderator variable	*N*	Coefficient	Regression coefficient (95% CIs)	*p*
Gender (percentage of females)	11	−0.09	(−0.39, 0.22)	0.537
Publication year	13	0.14	(−0.14, 0.42)	0.301
BDI at baseline	7	0.87	(0.2, 1.54)	**0.02**
Number of training sessions	13	0.01	(−0.25, 0.26)	0.959
Number of training trials per session	13	−0.27	(−0.65, 0.11)	0.146

Bold value means *p* < 0.05.

###### 3.4.1.1.2. Publication bias

**Figure 5:**
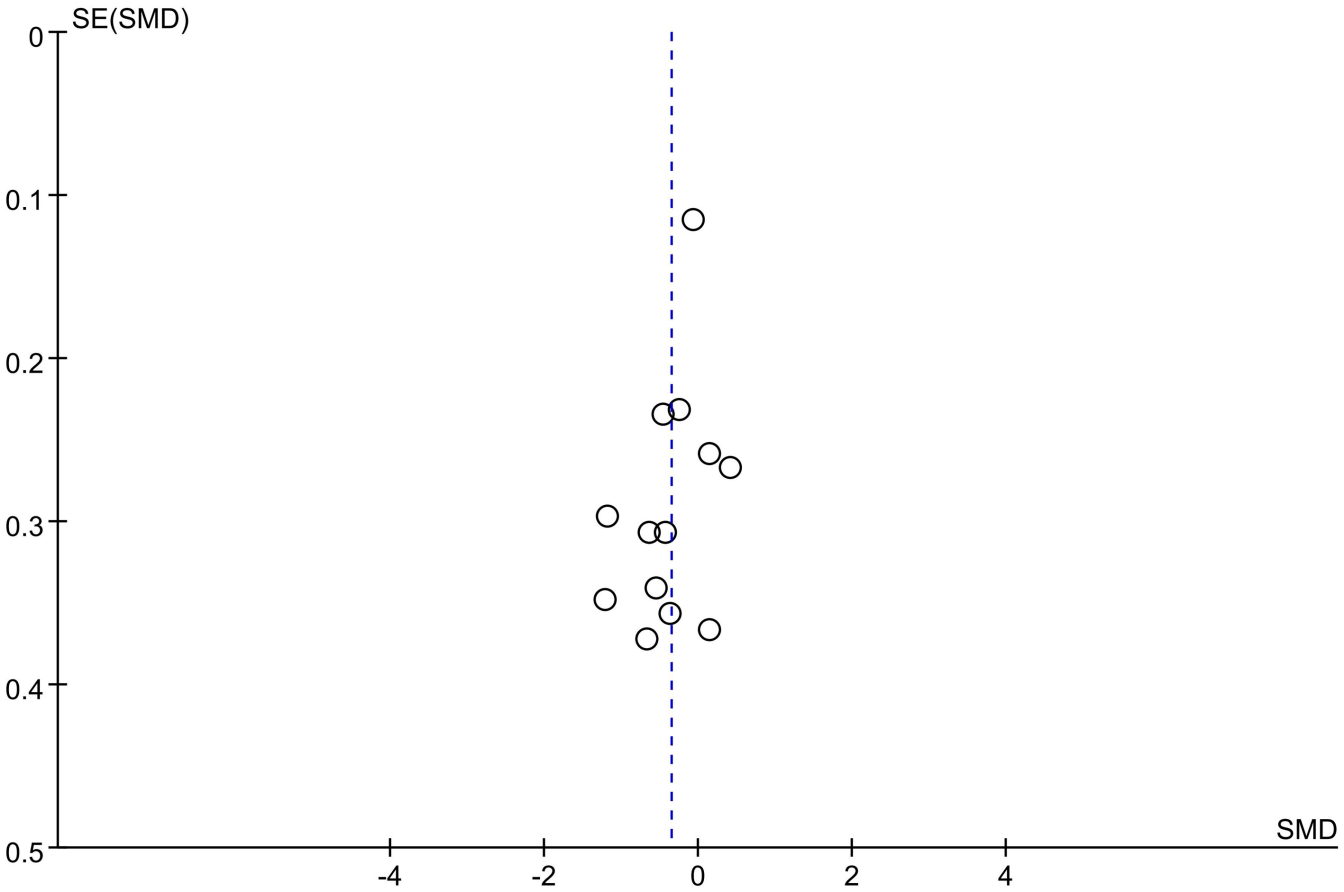
Funnel plot of depression outcome in comparison with ABM versus ACT.

The funnel plot was asymmetry, which indicated publication bias existed (Figure 5).

##### 3.4.1.2. ABM plus CT versus ACT plus CT

No difference was identified between ABM plus CT and ACT plus CT in alleviating depressive symptoms (SMD = −0.11, 95% CI −0.43 to 0.21), *I^2^* = 0%) (43, 49).

##### 3.4.1.3. ABM plus CT versus CT

Liu et al. (44) revealed that ABM plus CT had a better improvement in depressive symptoms than CT (*p* < 0.05).

#### 3.4.2. Secondary outcomes

##### 3.4.2.1. Rumination (ABM versus ACT)

**Figure 6:**
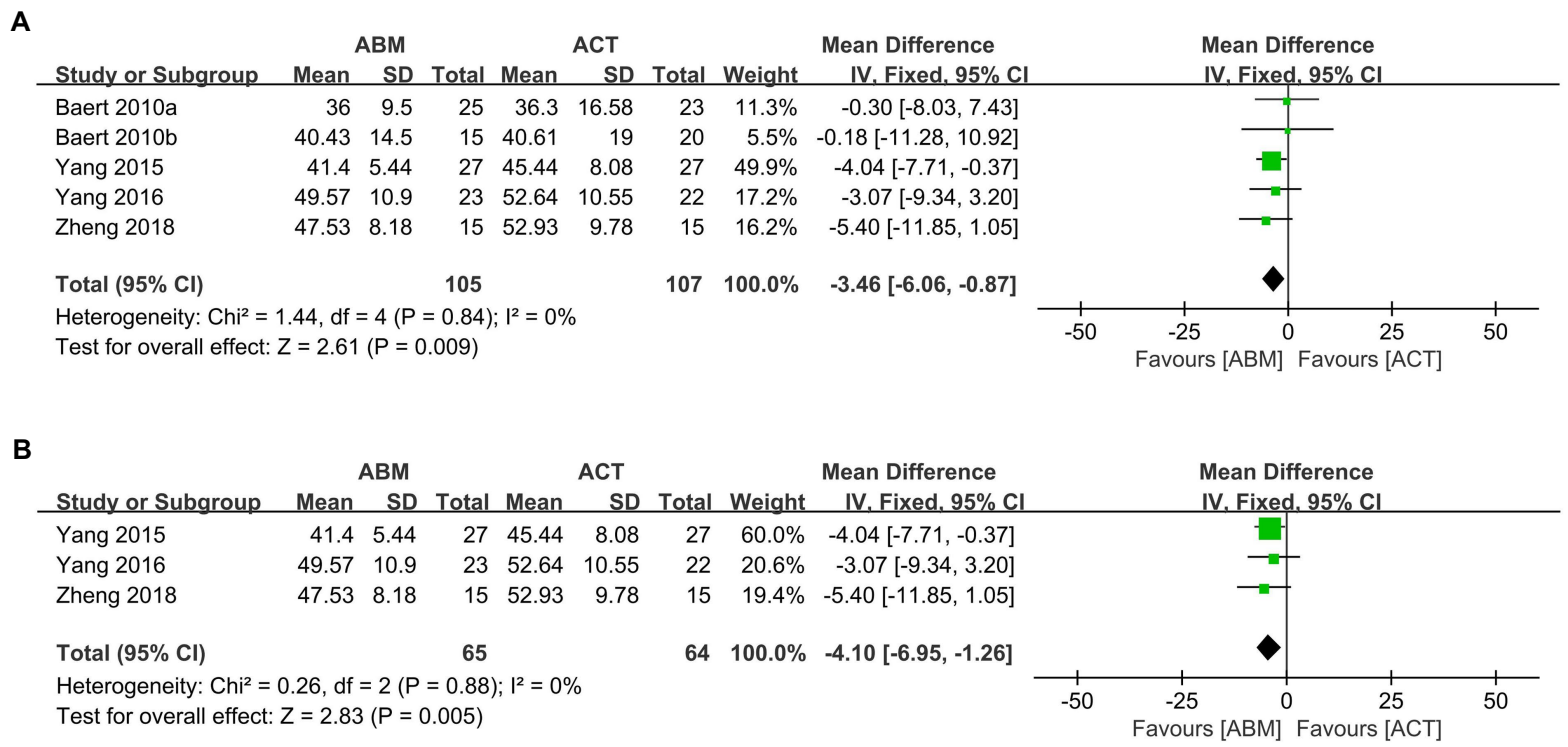
**(A)** Forest plot of rumination outcome in comparison with ABM versus ACT. **(B)** Forest plot of rumination outcome in comparison with ABM versus ACT after removing high risk-of-bias studies.

Four trials (17, 18, 37, 51) involving five datasets with 212 participants compared the effects of ABM with ACT for rumination. We found ABM was superior to ACT in relieving ruminative symptoms of patients with depression (MD = −3.46, 95% CI −6.06 to −0.87, *I^2^* = 0%; Figure 6A). According to sensitivity analysis, the results of rumination remained unchanged after excluding 2 datasets with a high risk of bias (37) (MD = −4.10, 95% CI −6.95 to −1.26, *I^2^* = 0%; Figure 6B).

##### 3.4.2.2. Attention control ability (ABM versus ACT)

**Figure 7:**
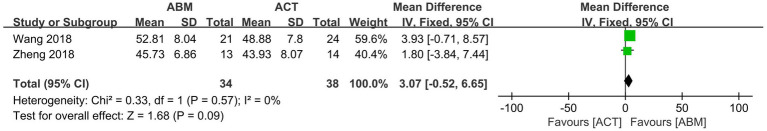
Forest plot of attention control outcome in comparison with ABM versus ACT.

According to two studies (48, 51), ABM did not differ from ACT in improving attention control scores (MD = 3.07, 95% CI −0.52 to 6.65), *I^2^* = 0%; Figure 7).

### 3.5. Certainty of the evidence

The results of the GRADE are shown in Supplementary Appendix 4. The certainty of the evidence of depression (ABM plus CT versus ACT plus CT) was graded as “moderate,” and the rest outcomes were considered as low or very low. The reasons for downgrading were mainly attributed to the risk of bias of included studies and imprecision and publication bias generated by small sample sizes.

## 4. Discussion

### 4.1. The effect of ABM on depression

Due to limited RCTs, previous meta-analyses demonstrated that ABM had no effect on relieving depressive symptoms (23, 24, 53). In the present meta-analysis, with 20 RCTs included, the synthesized data indicated that ABM might be an effective treatment for depressive disorder. Neurophysiologic studies have confirmed that ABM could modify functional brain connectivity within neural networks related to attentional control (54, 55). Specifically, Beaver’s study (56) found that ABM could reduce negative attention bias and enhance connectivity between the middle frontal gyrus and the dorsal anterior cingulate cortex (ACC). The dorsal ACC involves in attention control through connections with other frontal regions and plays an important role in the cognitive regulation of emotional information. Another study highlighted that greater activation in the lateral prefrontal cortex (PFC) and rostral ACC was observed when the direction of patients’ attention was opposite to ABM training (54). Hakamata et al. discovered that ABM enhanced the pulvinar control over the ventral frontoparietal network (vFPN) to maintain endogenous attention to behavioral targets and diminished the information exchanges between the postcentral gyrus and vFPN to inhibit the capture of exogenous attention by potential threats (57). Moreover, ABM could increase the levels of cortisol awakening responses, which were related to the development and progression of depression (58). Nevertheless, the mechanism of ABM for depression needs further exploration.

### 4.2. The effect of ABM on rumination

Significant reduction in rumination after ABM treatment was noted in our study. Depressive rumination is defined as a maladaptive emotion regulation strategy, which focuses one’s attention on sad mood and negative thoughts (59). The current study revealed that rumination was associated with negative attention bias and attentional control deficits with depression (60). Nolen-Hoeksema et al. (61) conducted a 3-year follow-up visit of 82 patients with depression and found individuals who engaged in rumination were more likely to develop depressive disorders. In addition, several studies concluded that ABM could decrease maladaptive ruminative processing by reducing negative attention bias, thus producing antidepressant effects (17, 62, 63). These findings suggested that ABM was able to promote resilience to the normal pattern of emotional regulation in depression by reducing rumination.

### 4.3. The effect of ABM on attentional control

Attentional control is a type of cognitive control schema and defined as the effortful allocation of attention toward goal-relevant information in the face of conflicting prepotent attentional demands (64). Evidence showed that patients with depression manifested hypoactivation in cortical structures of attentional control, which might be related to the impairment of cognitive performance (65). Attentional control appears to have an impact on depressive symptoms through rumination, and poor mood states can be regulated by improving attention control performance (65). Previous studies discovered that ABM might enhance attentional performance through the repetitive activation of neural circuitry with information processing and attentional control (62). However, based on limited studies, the results of our data showed that ABM was not effective to improve attentional control. Wang et al. (48) interpreted that long material presentation times may cause patients with depression to induce attentional avoidance toward negative stimuli in the later stages of attention processing. Therefore, the effect of ABM on attention control requires further investigation.

### 4.4. The protocol of ABM on depression

According to subgroup analysis, ABM training with the dot-probe task was more effective than ACT in reducing depression scores. It is reported that the dot probe has increasingly become an optimal type for attentional modulation (66, 67). However, Robert et al. (68) argued that the dot-probe task was not reliable in measuring reaction time, thus limiting its application in clinical practice. Future studies should identify the reliability of the dot-probe task for depression and compare the effect of different ABM tasks.

Our studies showed that ABM using training direction presented by left–right had a larger effect, while those using top-bottom training did not. Heeren et al. (69) explained that it was more ecologically relevant than processing faces presented horizontally rather than vertically. In contrast, Hakamata et al. (19) and Beard et al. (70) found that top-bottom training had a better effect than those with left–right. Different ABM protocols such as stimuli types or stimuli presentation time may be the reasons for the inconsistent findings.

Regarding training target stimuli, ABM using face stimulus was superior to those using word stimulus in our study. Similarly, Browning et al. (58) found that ABM training with face stimulus reduced the risk of depression, while ABM with word stimulus did not produce such beneficial effects. Jones et al. (71) revealed that ABM studies benefited more from using word stimulus combined with top-down training than those using face stimulus combined with left–right training.

ABM training delivered in the laboratory tended to yield a larger effect than those conducted at home, which was consistent with previous studies (23, 24, 69). Heeren et al. (69) inferred that patients who received ABM training in the standardized laboratory were less susceptible to outside interference.

Of note, ABM appeared to be beneficial for depressive adults and had no effect on adolescents. However, Hang et al. (10) concluded that younger participants could benefit more from ABM as they have a greater potential for attention control. In addition, it has been reported that the cognitive abilities of normal adults may decline in their 20s and 30s (72). Since few studies pay attention to depressive adolescents, more studies are needed to verify this finding.

In addition, the results of meta-regression also showed that BDI scores at baseline were the influencing factor of ABM, and lower BDI scores at baseline yielded a larger effect. Li et al. (73) found that severely depressive individuals exhibited deficits in executive function and attention compared to those with mild depression. A neuroimaging study showed that severe patients with depression manifested dorsolateral prefrontal cortex hypoactivity during attention control (74). This may elucidate why depressive patients with lower BDI benefit more from ABM.

As mentioned earlier, task types, target stimuli, training directions, training settings, age, and BDI scores at baseline were closely associated with the effect of ABM on depression. However, due to limited studies, the optimal protocols and potential influencing factors of ABM for patients with depression were undetermined, and more rigorously designed RCTs are needed to address these issues.

### 4.5. Deviations from the protocol

(1) Comparators mentioned in the protocol were sham ABM alone or sham ABM plus conventional rehabilitation or any other active intervention. In this review, participants in the control group received ACT alone, ACT plus CT, or CT alone. Sham ABM and ACT mean the same thing, while the ACT was widely used in the literature (10, 75); thus, we used ACT. (2) The age of the included patients was different. In the protocol, all patients over 18 years of age were included. Previous studies showed that ABM was also widely used for depression in adolescents (18, 40). To enlarge the applicability of this study, we did not impose age restrictions in this review. In addition, we conducted a subgroup analysis by different age groups to identify the advantages of ABM for specific age groups. (3) Different from the protocol, rumination and attentional control were added as secondary outcomes in the review. Rumination and attentional control are closely related to the occurrence and development of depression. The addition of secondary outcomes would facilitate a more broad and more comprehensive exploration of the effect of ABM on depression.

### 4.6. Limitations of this study

There were several potential limitations in our study. First, high heterogeneity was detected among the included studies; although the subgroup and meta-regression analyses were carried out, we still failed to find out the source of heterogeneity. Second, the risk of bias in most included studies was some concerns, and most of the evidence was low or very low certainty evidence, the findings should be taken with caution. Third, we included studies published in both Chinese and English, and publication bias might exist.

## 5. Conclusion

Due to high heterogeneity and limited studies, not enough current evidence supported that ABM could be an effective intervention to relieve depressive symptoms. More rigorous RCTs are required to verify the benefits and to explore the optimal protocols of ABM training for depression.

## Data availability statement

The original contributions presented in the study are included in the article/Supplementary material, further inquiries can be directed to the corresponding authors.

## Author contributions

H-sX, Y-xL, and Q-yZ designed the protocol and drafted the manuscript. J-xH, JL, and R-jJ revised this manuscript. D-lZ, X-bL, X-yG, JZ, JF, and YZ screened the articles, extracted data, and conducted data synthesis. JL and S-cA highlighted the research question and guided the whole process of this review. All authors contributed to the article and approved the submitted version.

## Funding

This study was supported by the National Key Research and Development Project of China (2019YFC1710302), the Key Project of Sichuan Province Science and Technology (2020YFS0284), the National Natural Science Foundation of China (81873354), and the Sichuan Province Science and Technology Program (2023NSFSC1824).

## Conflict of interest

The authors declare that the research was conducted in the absence of any commercial or financial relationships that could be construed as a potential conflict of interest.

## Publisher’s note

All claims expressed in this article are solely those of the authors and do not necessarily represent those of their affiliated organizations, or those of the publisher, the editors and the reviewers. Any product that may be evaluated in this article, or claim that may be made by its manufacturer, is not guaranteed or endorsed by the publisher.

## Footnotes

^1^ https://www.crd.york.ac.uk/prospero/display_record.php?ID=CRD42021279163

^2^ClinicalTrials.gov and www.chictr.org.cn
