# Supplementary material for: Attention bias modification for depression: A systematic review and meta-analysis
Source: Front Psychiatry. 2023 Mar 10;14:1098610. doi: 10.3389/fpsyt.2023.1098610 (PMC10036757; doi:10.3389/fpsyt.2023.1098610)
Supplement: Supplementary file 1 [file Data_Sheet_1.zip › Supplementary Appendix/Appendix 2 Search strategies.docx]

**Search strategies of electronic databases**

| **Database** | **Search strategy** |
| --- | --- |
| Pubmed | #1 Depression [MeSH]  #2 Depressive disorder, major [MeSH]  #3 Depress*[tiab] OR MDD[tiab] OR Depressive syndrome*[tiab] OR Depressive neuroses[tiab] OR Endogenous depression*[tiab] OR Unipolar depression*[tiab] OR Dysthymi*[tiab] OR Dysthymic disorder[tiab]  #4 #1 OR #2 OR #3  #5 Attention* bias modification[tiab]  #6 Attention* training[tiab]  #7 ABM[tiab]  #8 #5 OR #6 OR #7  #9 #4 AND #8 |
| Embase | #1 'depression'/exp OR 'major depression'/exp  #2 depress*:ti,ab,kw OR mdd:ti,ab,kw OR 'depressive syndrome*':ti,ab,kw OR 'depressive neuroses':ti,ab,kw OR 'endogenous depression*':ti,ab,kw OR 'unipolar depression*':ti,ab,kw OR dysthymi*:ti,ab,kw OR 'dysthymic disorder':ti,ab,kw  #3 #1 OR #2  #4 'attention* bias modification':ti,ab,kw OR 'attention* training':ti,ab,kw OR abm:ti,ab,kw  #5 #3 and #4 |
| Cochrane Library | #1 MeSH descriptor: [Depression] explode all trees OR MeSH descriptor: [Depressive Disorder, Major] explode all trees  #2 (Depress*):ti,ab,kw OR (MDD):ti,ab,kw OR (Depressive syndrome*):ti,ab,kw OR (Depressive neuroses):ti,ab,kw OR (Endogenous depression*):ti,ab,kw OR (Unipolar depression*):ti,ab,kw OR (Dysthymi*):ti,ab,kw OR (Dysthymic disorder):ti,ab,kw  #3 #1 OR #2  #4 (Attention* bias modification):ti,ab,kw OR (Attention* training):ti,ab,kw OR (ABM):ti,ab,kw  #5 #3 and #4 |
| CNKI | (SU = '抑郁' OR SU = '忧郁') AND (SU = '注意偏向矫正' OR SU = '注意偏向训练') |
| Wanfang | 题名或关键词: (("抑郁" or "忧郁") and ("注意偏向矫正" or "注意偏向训练") |
| VIP | (题名或关键词=抑郁OR忧郁) AND (题名或关键词=注意偏向矫正OR注意偏向训练) |
| CBM | [("抑郁"[常用字段:智能] OR "忧郁"[常用字段:智能])](javascript:toDoRelimitSearch();) AND ("注意偏向矫正"[常用字段:智能] OR "注意偏向训练"[常用字段:智能]) |
