# Supplementary material for: Attention bias modification for depression: A systematic review and meta-analysis
Source: Front Psychiatry. 2023 Mar 10;14:1098610. doi: 10.3389/fpsyt.2023.1098610 (PMC10036757; doi:10.3389/fpsyt.2023.1098610)
Supplement: Supplementary file 1 [file Data_Sheet_1.zip › Supplementary Appendix/Appendix 3 The list of excluded records.docx]

**A list of excluded studies by reading full text.**

**Unavailable data:**

Beevers CG, Clasen PC, Enock PM, Schnyer DM. Attention bias modification for major depressive disorder: Effects on attention bias, resting state connectivity, and symptom change. J Abnorm Psychol. 2015 Aug;124(3):463-75. doi: 10.1037/abn0000049.

Beevers CG, Hsu KJ, Schnyer DM, Smits JAJ, Shumake J. Change in negative attention bias mediates the association between attention bias modification training and depression symptom improvement. J Consult Clin Psychol. 2021 Oct;89(10):816-829. doi: 10.1037/ccp0000683.

Blaut A, Paulewicz B, Szastok M, Prochwicz K, Koster E. Are attentional bias and memory bias for negative words causally related? J Behav Ther Exp Psychiatry. 2013 Sep;44(3):293-9. doi: 10.1016/j.jbtep.2013.01.002.

Browning M, Holmes EA, Charles M, Cowen PJ, Harmer CJ. Using attentional bias modification as a cognitive vaccine against depression. Biol Psychiatry. 2012 Oct 1;72(7):572-9. doi: 10.1016/j.biopsych.2012.04.014.

Duque A, Vazquez C. A failure to show the efficacy of a dot-probe attentional training in dysphoria: Evidence from an eye-tracking study. J Clin Psychol. 2018 Dec;74(12):2145-2160. doi: 10.1002/jclp.22652.

Everaert J, Mogoaşe C, David D, Koster EH. Attention bias modification via single-session dot-probe training: Failures to replicate. J Behav Ther Exp Psychiatry. 2015 Dec;49(Pt A):5-12. doi: 10.1016/j.jbtep.2014.10.011.

Ferrari GR, Möbius M, van Opdorp A, Becker ES, Rinck M. Can't Look Away: An Eye-Tracking Based Attentional Disengagement Training for Depression. Cognit Ther Res. 2016;40(5):672-686. doi: 10.1007/s10608-016-9766-0.

Kruijt AW, Putman P, Van der Does W. The effects of a visual search attentional bias modification paradigm on attentional bias in dysphoric individuals. J Behav Ther Exp Psychiatry. 2013 Jun;44(2):248-54. doi: 10.1016/j.jbtep.2012.11.003.

LeMoult J, Joormann J, Kircanski K, Gotlib IH. Attentional bias training in girls at risk for depression. J Child Psychol Psychiatry. 2016 Nov;57(11):1326-1333. doi: 10.1111/jcpp.12587.

Li H, Wei D, Browning M, Du X, Zhang Q, Qiu J. Attentional bias modification (ABM) training induces spontaneous brain activity changes in young women with subthreshold depression: a randomized controlled trial. Psychol Med. 2016 Apr;46(5):909-20. doi: 10.1017/S003329171500238X.

**No RCT:**

Cooper JA, Gorlick MA, Denny T, Worthy DA, Beevers CG, Maddox WT. Training attention improves decision making in individuals with elevated self-reported depressive symptoms. Cogn Affect Behav Neurosci. 2014 Jun;14(2):729-41. doi: 10.3758/s13415-013-0220-4.

Lazarov A, Ben-Zion Z, Shamai D, Pine DS, Bar-Haim Y. Free viewing of sad and happy faces in depression: A potential target for attention bias modification. J Affect Disord. 2018 Oct 1;238:94-100. doi: 10.1016/j.jad.2018.05.047.

Li M. Attentional biases and a functional MRI study of depressed patients -aIso a discussion on attention training in depression

Liu H N. Impacts of attentional training on attention bias of sub-clinical depressed undergraduates. Chinese Journal of Behavioral Medical Science. 2016,25(1):60-64. doi:10.3760/cma.j.issn.1674-6554.2016.01.013.

Papageorgiou C, Wells A. Treatment of recurrent major depression with attention training. Cognitive and Behavioral Practice. 2000,7(4):407-413. doi:10.1016/S1077-7229(00)80051-6.

Tsumura H, Shimada H, Nomura K, Sugaya N, Suzuki K. The effects of attention retraining on depressive mood and cortisol responses to depression-related stimuli1 [J]. Japanese Psychological Research, 2012, 54(4): 400-11. [doi:10.1111/j.1468-5884.2012.00523.x](https://doi.org/10.1111/j.1468-5884.2012.00523.x)

**Repeated publication：**

Ding Z R. Attention bias modification treatment for adolescent major depression: a first randomized controlled trial. Hunan Normal University, 2015. doi:10.7666/d.Y2802309.

Hilland E, Landrø NI, Harmer CJ, Browning M, Maglanoc LA, Jonassen R. Attentional bias modification is associated with fMRI response toward negative stimuli in individuals with residual depression: a randomized controlled trial. J Psychiatry Neurosci. 2020 Jan 1;45(1):23-33. doi: 10.1503/jpn.180118.

Hilland E, Landrø NI, Harmer CJ, Maglanoc LA, Jonassen R. Within-Network Connectivity in the Salience Network After Attention Bias Modification Training in Residual Depression: Report From a Preregistered Clinical Trial. Front Hum Neurosci. 2018 Dec 21;12:508. doi: 10.3389/fnhum.2018.00508.

Jonassen R, Harmer CJ, Hilland E, Maglanoc LA, Kraft B, Browning M, Stiles TC, Haaland VØ, Berge T, Landrø NI. Effects of Attentional Bias Modification on residual symptoms in depression: a randomized controlled trial. BMC Psychiatry. 2019 May 8;19(1):141. doi:10.1186/s12888-019-2105-8.

Wang, M.L. The long-term efficacy of attention bias modification treatment for individuals with depressive disorders:a randomized double-blind controlled trial. Hunan Normal University, 2020. doi:10.27137/d.cnki.ghusu.2020.002251.

Zeng K B. Neutral and positive attention bias training improves depressive symptoms and the comparison of the role of redundant thought. Hunan Normal University, 2018.

**Conference abstracts**

Xu X L, Chen L S. An experimental study of the positive attention training alleviating college students,depression. The 18th National Conference on Psychology. 2015(3):155-157. DOI:10.3969/j.issn.1008-4916.2015.03.050.

Yang W H, Wang M L, Huang B Y, Zheng Z F, Zhang C G.The Long-term Efficacy of Attention Bias Modification Treatment for Individuals with Depressive Disorders:A Randomized Double-blind Controlled Trial. The 22nd National Conference on Psychology. 2019:731-733.
