# Supplementary material for: Attention bias modification for depression: A systematic review and meta-analysis
Source: Front Psychiatry. 2023 Mar 10;14:1098610. doi: 10.3389/fpsyt.2023.1098610 (PMC10036757; doi:10.3389/fpsyt.2023.1098610)
Supplement: Supplementary file 1 [file Data_Sheet_1.zip › Supplementary Appendix/Appendix 4 Grade-ABM.docx]

**Quality of evidence per outcome from selected studies by the GRADE approach**

| **Quality assessment** | | | | | | | **No of patients** | | **Effect** | | **Quality** | **Importance** |
| --- | --- | --- | --- | --- | --- | --- | --- | --- | --- | --- | --- | --- |
|  |  |  |  |  |  |  |  |  |  |  |  |  |
| **No of studies** | **Design** | **Risk of bias** | **Inconsistency** | **Indirectness** | **Imprecision** | **Other considerations** | **ABM** | **ACT** | **Relative (95% CI)** | **Absolute** |  |  |
| **Depression (ABM vs. ACT)** | | | | | | | | | | | | |
| 17 | randomised trials | serious | very serious | no serious indirectness | no serious imprecision | none | 529 | 526 | - | SMD 0.48 lower (0.80 to 0.17 lower) | ⊕OOO VERY LOW | CRITICAL |
| **Rumination (ABM vs. ACT)** | | | | | | | | | | | | |
| 5 | randomised trials | serious | no serious inconsistency | no serious indirectness | serious | none | 105 | 107 | - | MD 3.46 lower (6.06 to 0.87 lower) | ⊕⊕OO LOW | IMPORTANT |
| **Attention control (ABM vs. ACT)** | | | | | | | | | | | | |
| 2 | randomised trials | no serious risk of bias | no serious inconsistency | no serious indirectness | serious | reporting bias | 34 | 38 | - | MD 3.07 higher (0.52 lower to 6.65 higher) | ⊕⊕OO LOW | IMPORTANT |
| **Depression** **(ABM plus CT vs. ACT plus CT)** | | | | | | | | | | | | |
| 2 | randomised trials | no serious risk of bias | no serious inconsistency | no serious indirectness | serious | none | 77 | 77 | - | SMD 0.11 lower (0.43 lower to 0.21 higher) | ⊕⊕⊕O MODERATE | CRITICAL |
| **Depression (ABM plus CT vs. CT)** | | | | | | | | | | | | |
| 1 | randomised trials | serious | no serious inconsistency | no serious indirectness | serious | reporting bias | 26 | 27 | - | SMD 2.31 lower (4.11 lower to 0.51 lower) | ⊕OOO VERY LOW | CRITICAL |

Abbreviations: CI, confidence interval; SMD, standardized mean difference; MD, mean difference;
